# Causal Influence of Linguistic Learning on Perceptual and Conceptual Processing: A Brain-Constrained Deep Neural Network Study of Proper Names and Category Terms

**DOI:** 10.1523/JNEUROSCI.1048-23.2023

**Published:** 2024-02-28

**Authors:** Phuc T. U. Nguyen, Malte R. Henningsen-Schomers, Friedemann Pulvermüller

**Affiliations:** ^1^Brain Language Laboratory, Department of Philosophy and Humanities, Freie Universität Berlin, Berlin 14195, Germany; ^2^Cluster of Excellence “Matters of Activity Image Space Material”, Humboldt-Universität zu Berlin, Berlin 10099, Germany; ^3^Berlin School of Mind and Brain, Berlin 10099, Germany; ^4^Einstein Center for Neurosciences, Berlin D-10117, Germany

**Keywords:** category learning, concept formation, deep neural network, Hebbian associative learning, instance representation, verbal symbol learning

## Abstract

Language influences cognitive and conceptual processing, but the mechanisms through which such causal effects are realized in the human brain remain unknown. Here, we use a brain-constrained deep neural network model of category formation and symbol learning and analyze the emergent model’s internal mechanisms at the neural circuit level. In one set of simulations, the network was presented with similar patterns of neural activity indexing instances of objects and actions belonging to the same categories. Biologically realistic Hebbian learning led to the formation of instance-specific neurons distributed across multiple areas of the network, and, in addition, to cell assembly circuits of “shared” neurons responding to all category instances—the network correlates of conceptual categories. In two separate sets of simulations, the network learned the same patterns together with symbols for individual instances [“proper names” (PN)] or symbols related to classes of instances sharing common features [“category terms” (CT)]. Learning CT remarkably increased the number of shared neurons in the network, thereby making category representations more robust while reducing the number of neurons of instance-specific ones. In contrast, proper name learning prevented a substantial reduction of instance-specific neurons and blocked the overgrowth of category general cells. Representational similarity analysis further confirmed that the neural activity patterns of category instances became more similar to each other after category-term learning, relative to both learning with PN and without any symbols. These network-based mechanisms for concepts, PN, and CT explain why and how symbol learning changes object perception and memory, as revealed by experimental studies.

## Significance Statement

How do verbal symbols for specific individuals (*Micky Mouse*) and object categories (*house mouse*) causally influence conceptual representation and processing? Category terms and proper names (PN) have been shown to promote category formation and instance learning, potentially by directing attention to category critical and object-specific features, respectively. Yet the mechanisms underlying these observations at the neural circuit level remained unknown. Using a mathematically precise deep neural network model constrained by properties of the human brain, we show category-term learning strengthens and solidifies conceptual representations, whereas PN support object-specific mechanisms. Based on network internal mechanisms and unsupervised correlation-based learning, this work offers neurobiological explanations for the causal effects of symbol learning on concept formation, category building, and instance representation in the human brain.

## Introduction

Most signs and symbols are used to speak about objects and actions. This led philosophers and logicians to propose that the referential link between symbol and world is essential for meaning and semantics (Wittgenstein, 1922; Frege, 1948). Yet there are quite different relationships between symbols and their related real-world entities. One most essential difference exists between “proper names” (PN) used to speak about a single object or individual (e.g., “Mickey Mouse”) and “category terms” (CT), which can refer to members of an entire class or conceptual category (e.g., “house mouse”). Such differences between referential symbols are well-described at the semantic level, but not understood in terms of their underlying mechanisms in the mind and brain.

The need for mechanistic neurobiological models of symbols and their meaning comes from reports about the causal influences of language on perception, attention, and memory. It had long been speculated and recently been confirmed that, when human subjects learn words for objects, language may help humans to attend to and distinguish between them (Majid et al., 2004; Whorf and Carroll, 2007; Miller et al., 2018; Vanek et al., 2021). Experimental research in infants showed that learning “labels” for objects increases their attention to these objects (Baldwin and Markman, 1989), which further establishes an attention-catching function of language. However, this general insight requires further specification to capture the different effects of CT and PN. In particular, learning a new symbol for a category of objects makes infants attend to the shared features of these objects and facilitates their learning of the conceptual category (Gelman and Markman, 1986, 1987; Plunkett et al., 2008); the latter even holds if the objects show little perceptual similarity (Graham et al., 2013). On the other hand, the category building function of language is absent when object-specific PN are learned. In this case, the infant's attention is directed not toward the common category features of objects but to idiosyncratic and object-specific features instead (Scott and Monesson, 2009; LaTourrette and Waxman, 2020). In summary, category-term learning directs attention to shared features of objects (Waxman and Booth, 2001; Dewar and Xu, 2007; Althaus and Mareschal, 2014; Althaus and Plunkett, 2016), whereas unique proper name learning highlights idiosyncratic and object-specific features (Best et al., 2010; Barnhart et al., 2018; Pickron et al., 2018; LaTourrette and Waxman, 2020). These specific and replicable effects of PN and CT on perception and attention have been explained in terms of different “strategies” applied by the learner. A neurobiological explanation of why these specific effects occur is still missing.

**Figure 1.**
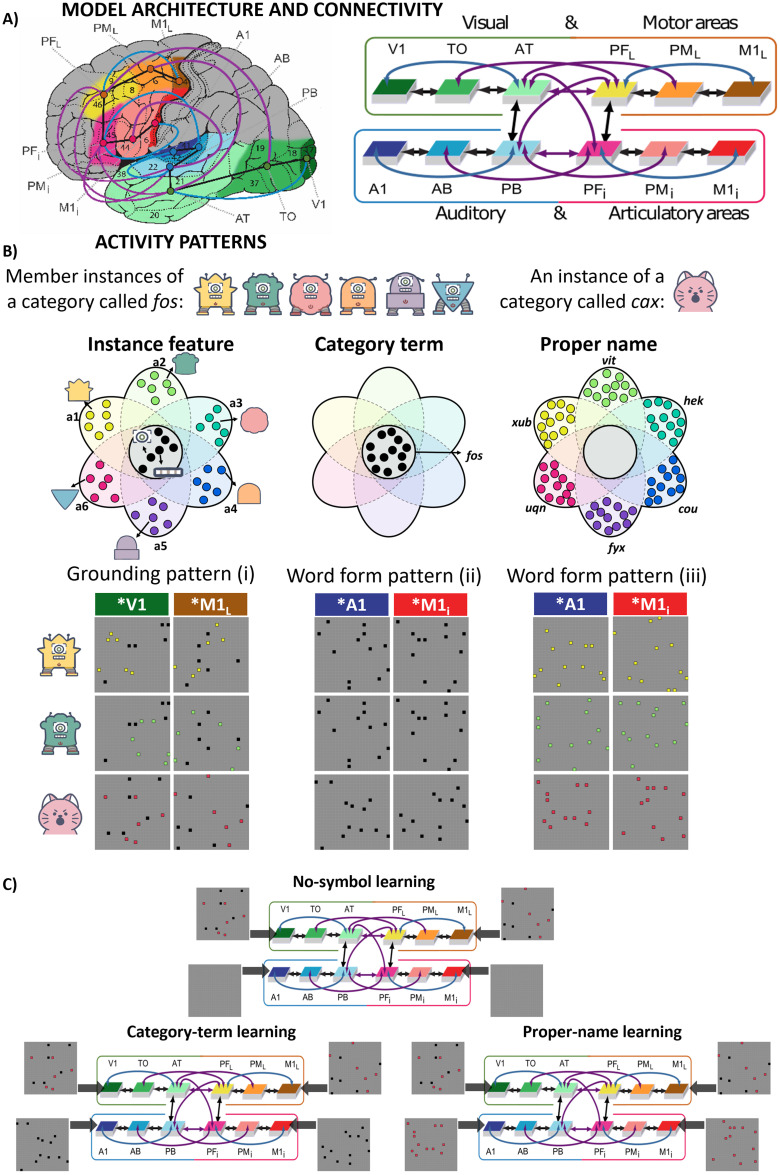
***A***, Area structure and between-area connectivity of the neural network model. Left: The network model's 12 cortical areas in the left fronto-temporo-occipital lobes—inferior frontal articulatory (red) and superior temporal auditory systems (blue) of the perisylvian areas and the lateral frontal hand motor system (yellow/orange/brown) and visual “what” stream (green) in the extrasylvian cortex. Right: Connections among the 12 modeled brain areas—direct connections between adjacent areas (black arrows), second nearest-neighbor areas (blue arrows), and long-distant links (purple arrows). Figure modified from Tomasello et al. (2018). ***B***, Schematic illustrations of activity patterns for instances of two categories. The categories are illustrated with images of robots and cat faces but note that this is for illustrative purposes. The actual input to the model was not images, but grounding patterns consisting of sets of activated neurons (see main text for details). Active neurons of given activity patterns were either shared among instances of the same category (black) or unique to each instance (color). Each model area included 25×25 excitatory neurons, i.e., 625 cells. Left: In grounding patterns (i) presented to *V1/*M1_L_, six shared active neurons (black) code for the common perceptual–semantic features of the category “a,” and six unique neurons (color) represent instance-specific perceptuomotor features from each of the category members. Member instances of one category activated the same six shared neurons while the instance from another category activated a different set of six shared neurons; each instance also activated six unique neurons. Middle: 12 neurons (black) make up word form pattern for the category term; in the category term condition, member instances coactivated with the same word form pattern (ii) in *A1/*M1_i_. Right: 12 unique neurons (color) represent each proper name of an individual instance, which are activated 1-to-1 with these instances in the proper name condition. Instances were coactivated with distinct different word form patterns (iii) in *A1/*M1_i_ regardless of category. ***C***, Simulating no-symbol learning (top), category term learning (bottom-left), and proper name learning (bottom-right) where no word form pattern, word form patterns (ii), and word form pattern (iii) were presented to *A1/*M1_i_, respectively.

**Figure 2.**
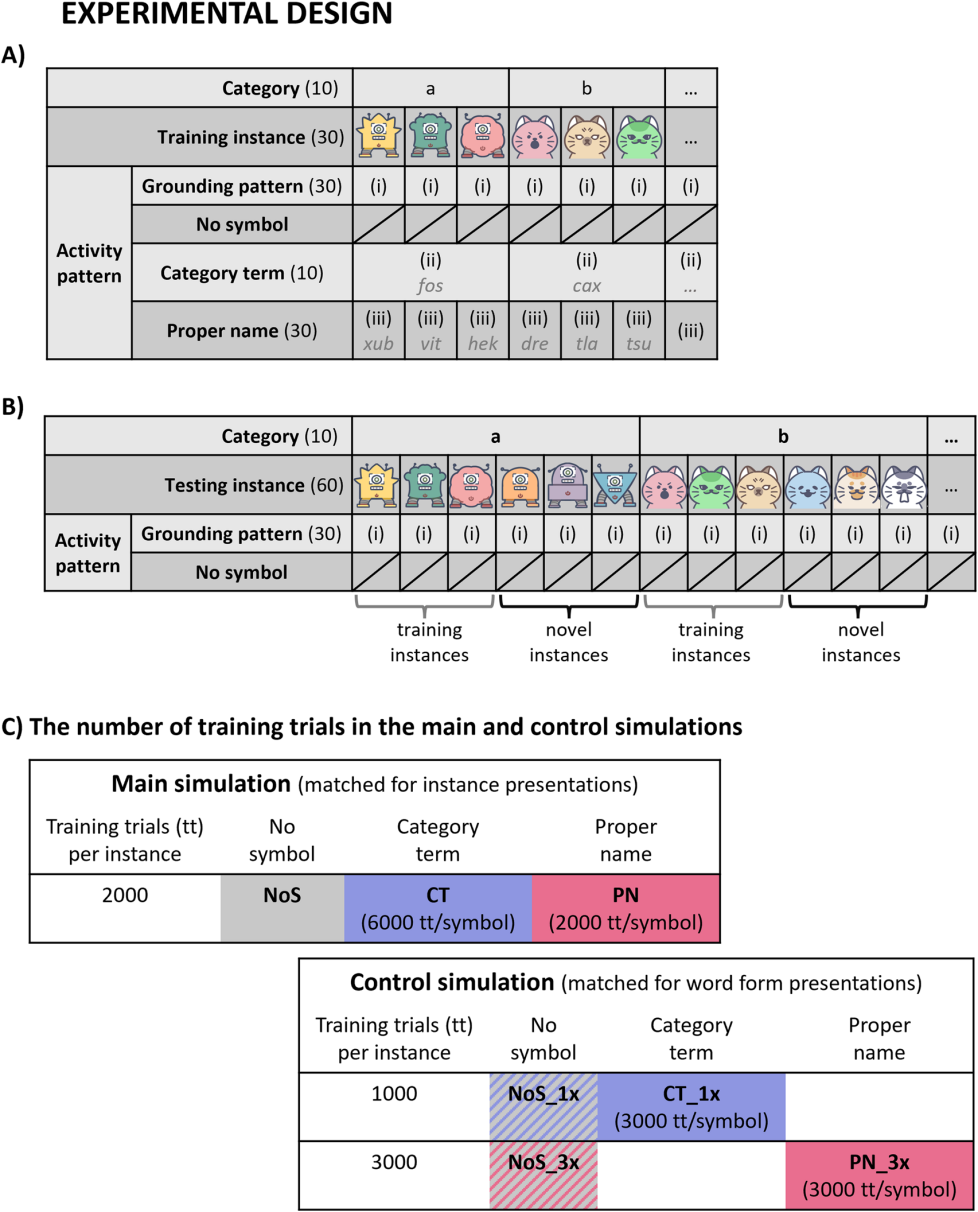
Experimental design used for instance learning and conceptual grounding. ***A***, Training phase with 30 object instances from ten categories. The categories are illustrated with images of robots and cat faces, but note that this is for illustrative purposes. The actual input to the model was not images, but grounding patterns consisting of sets of activated neurons (see main text for details). For each trained instance, the grounding pattern (i) was either presented to the network on its own (no symbol) or combined with a “word form pattern” of type (ii, category term) or type (iii, proper name). ***B***, Testing phase with a collection of the initially trained 30 instances and 30 novel instances from the 10 original categories, resulting in 60 testing instances (i.e., 6 per category). ***C***, Training conditions in the main simulations (top) and control simulations (bottom) differ in the number of training trials (tt) to match the number of instance representations and the number of word form representations, respectively.

Why and how can PN and CT direct attention to specific versus shared features of category members? To develop a mechanistic explanation, we used a brain-constrained deep neural network designed according to the area structure and connectivity of major areas relevant to language and conceptual processing (Garagnani et al., 2007; Tomasello et al., 2018; Pulvermüller et al., 2021). Six “areas” of the model simulated processes in superior temporal and inferior frontal perisylvian language areas and six extrasylvian model areas simulated inferior temporo-occipital visual “where” processing stream and dorsolateral prefrontal and motor cortices (Fig. 1*A*). In the no-symbol (NoS) condition, the model learned activity patterns each representing 1 of 60 instances of objects or actions belonging to 10 different categories. In learning-with-symbols conditions, the model learned additional activity patterns representing word forms of PN or CT (Figs. 1*B,C*, 2*A*). After learning, the model was tested by activating previously trained instance patterns of each category and, in addition, new patterns for novel instances belonging to the same categories (Fig. 2*B*). We documented the neural and cognitive effects of PN and CT on instance and category learning in the model. In-depth analyses of the emerging activation patterns and representations were provided by using representational similarity analysis (RSA; Kriegeskorte et al., 2008) and by classifying neurons into instance-specific and category general ones.

## Materials and Methods

### Participants

The current work does not contain experiments with human participants or animal subjects.

#### Neurobiological constraints

In contrast to many neural network models, the brain-constrained model aimed at biological plausibility by applying a range of structural and functional constraints (used in these studies Pulvermüller and Garagnani, 2014; Tomasello et al., 2018; Henningsen-Schomers and Pulvermüller, 2022; for review, see Pulvermüller et al., 2021) realizing:

1. neurophysiological dynamics of spiking pyramidal cells (Connors et al., 1982; Matthews, 2001),
2. synaptic weights under the modification of unsupervised Hebbian-type learning (i.e., synaptic plasticity and learning were modified according to the biologically plausible unsupervised Hebbian principles that incorporated both long-term potentiation and long-term depression; Artola and Singer, 1993),
3. local and global activity regulation (Braitenberg, 1978; Yuille and Geiger, 1995) based on local and area-specific inhibition mechanisms (Knoblauch and Palm, 2002),
4. excitatory and inhibitory within-area local connectivity (including sparse, random, and initially weak excitatory links whose probability falls off with distance; Kaas, 1997; Braitenberg and Schüz, 1998),
5. between-area global connectivity built on neuroanatomical evidence, and
6. built-in uncorrelated white noise in neurons of (1) all areas during training and testing mimicked spontaneous baseline neuronal firing and (2) additional noise in neurons of areas not stimulated by patterns during training, which simulated uncorrelated sensory or motor activity unrelated to instances or symbols (Rolls and Deco, 2010).

Table 2 supplies the model specifications and parameters chosen in this current work.

### Model description

We applied a brain-constrained deep neural network model including spiking model neurons and 12 model areas to model sensorimotor, conceptual, and linguistic mechanisms in the left-hemispheric language-dominant fronto-temporo-occipital regions of the human brain, as described in previous studies by Tomasello et al. (2018) and Henningsen-Schomers and Pulvermüller (2022).

#### Anatomical architecture and connectivity

To distinguish between subparts of neural networks from their target cortical structures of the real human brain, all model areas are marked by an asterisk before (e.g., *A1, *V1). The architecture modeled three areas representing the ventral visual system [i.e., primary visual cortex (*V1), temporo-occipital area (*TO), anterior–temporal area (*AT)] and three areas representing the dorsolateral action system [i.e., dorsolateral fronto-central motor (*M1_L_), premotor cortex (*PM_L_), prefrontal cortex (*PF_L_)]. These formed the extrasylvian region for sensorimotor processing where semantic information was stored. Another six areas of the perisylvian region for word form processing housed articulatory–phonological and acoustic–phonological information. These areas involved the three areas of the auditory system [i.e., primary auditory cortex (*A1), auditory belt (*AB), parabelt areas (*PB)] and three inferior frontal articulatory and prefrontal areas [i.e., inferior primary motor cortex (*M1_i_), premotor cortex (*PM_i_), prefrontal cortex (*PF_i_)], respectively. Between-area connections were reciprocal and connected next-neighbor areas, second next neighbors (Schomers et al., 2017), and long-distance corticocortical links supported by neuroanatomical evidence in the literature (Table 1).

**Table 1.** Connectivity structure of the modeled cortical areas with neuroanatomical evidence

Modeled areas	References
**Between-area connectivity (black arrows)**	
Perisylvian system	
A1, AB, PB	Pandya and Yeterian, 1985; Pandya, 1995; Rauschecker and Tian, 2000
PF_i_, PM_i_, M1_i_	Pandya and Yeterian, 1985; Young et al., 1995a,b
Extrasylvian system	
V1, TO, AT	Bressler et al., 1993; Distler et al., 1993
PF_L_, PM_L_, M1_L_	Pandya and Yeterian, 1985; Arikuni et al., 1988; Lu et al., 1994; Rizzolatti and Luppino, 2001; Dum and Strick, 2002, 2005
Between system	
AT, PB	Gierhan, 2013
PF_i_, PF_L_	Yeterian et al., 2012
**Long-distance corticocortical connections (purple arrows)**	
Perisylvian system	
PF_i_, PB	Meyer et al., 1999; Romanski et al., 1999a,b; Paus et al., 2001; Catani et al., 2005; Parker et al., 2005; Rilling et al., 2008; Makris and Pandya, 2009
PB, PM_i_	Rilling et al., 2008; Saur et al., 2008
AB, PF_i_	Romanski et al., 1999a,b; Kaas and Hackett, 2000; Petrides and Pandya, 2009; Rauschecker and Scott, 2009
Extrasylvian system	
AT, PF_L_	Bauer and Jones, 1976; Fuster et al., 1985; Ungerleider et al., 1989; Eacott and Gaffan, 1992; Webster et al., 1994; Parker and Gaffan, 1998; Chafee and Goldman-Rakic, 2000
AT, PM_L_	Bauer and Fuster, 1978; Fuster et al., 1985; Pandya and Barnes, 1987; Seltzer and Pandya, 1989; Chafee and Goldman-Rakic, 2000
TO, PF_L_	Bauer and Jones, 1976; Fuster and Jervey, 1981; Fuster et al., 1985; Seltzer and Pandya, 1989; Makris and Pandya, 2009
Between systems	
PB, PF_L_	Pandya and Barnes, 1987; Romanski et al., 1999a,b
AT, PF_i_	Pandya and Barnes, 1987; Ungerleider et al., 1989; Webster et al., 1994; Romanski, 2007; Petrides and Pandya, 2009; Rilling, 2014
**Second next-neighbor “jumping” links (blue arrows)**	
Perisylvian system (Rilling et al., 2008, 2012; Thiebaut de Schotten et al., 2012; Rilling and van den Heuvel, 2018)	
A1, PB	Pandya and Yeterian, 1985; Young et al., 1994
PF_i_, M1_i_	Deacon, 1992; Young et al., 1995b; Guye et al., 2003
Extrasylvian system (Thiebaut de Schotten et al., 2012)	
V1, AT	Catani et al., 2003; Wakana et al., 2004
PF_L_, M1_L_	Deacon, 1992; Young et al., 1995a; Guye et al., 2003

Table taken from Tomasello et al. (2018).

In the current neural network model, the fundamental information processing units are artificial neuron-like elements or cells. Each modeled area comprised two layers of 625 e-cells and 625 i-cells that mimicked an (excitatory) pyramidal spiking neuron and a cluster of (inhibitory) interneurons hosted within the same cortical column in the cortical area. A more elaborate description of the firing behavior of such neurons can be found in the studies of Garagnani et al. (2017), Tomasello et al. (2018), and Henningsen-Schomers and Pulvermüller (2022).

### Activity patterns applied to the networks

A total of 60 “grounding patterns” were defined as sensorimotor activation patterns thought to represent specific sensory-motor experiences of 60 different objects or “instances.” Groups of six instances overlapped in their neuronal grounding patterns and were taken as representations of different instances of the same concept (e.g., different robots). Note that the images of robots and cat faces for category members are to be taken purely for illustrative purposes here—the actual training patterns of the models consisted of sets of activated neurons with no systematic relationship to images of robots or cat faces. A category comprised three trained instances and three novel instances not presented during training; all six instance patterns were used for network testing (Fig. 2*A,B*). Each category instance was neuronally coded as a set of perceptual and motor neuron activations in the primary visual and hand motor areas of the brain-constrained network. These instance-related grounding patterns were activated either on their own or together with additional patterns of neuronal activation in the network's articulatory and auditory cortices, which were thought to implement symbol forms, that is, verbal labels or spoken word forms. These “word form patterns” were used either as PN and therefore specifically with only one grounding pattern or as CT, and therefore the same word form pattern co-occurred with all three trained grounding patterns of one category. To control the effect of nonlinguistic factors, a third class of trained grounding patterns was learned without concordant auditory–articulatory activation. Thus, we generated three classes of simulated stimulation patterns: (i) instance-related grounding patterns applied to *V1/*M1_L_ (Fig. 1*B*, left), (ii) category term patterns to *A1/*M1_i_ (Fig. 1*B*, middle), and (iii) proper name patterns to *A1/*M1_i_ (Fig. 1*B*, right). Sensorimotor experiences of instances were simulated with conceptual grounding patterns (i), and symbol-related auditory–articulatory activity was simulated using word form patterns (ii and iii).

For visualization and a better conceptual understanding of the use of activity patterns, see Figure 1*B,C*. Instances belonging to the same category were simulated by similar grounding patterns, following Henningsen-Schomers and Pulvermüller (2022): within-category instances had grounding patterns that shared 50% of their feature neurons and differed from each other in the other half; grounding patterns simulating instances from different categories had no neuronal overlap. For each grounding pattern (i), a subset of 12 out of 625 potential cells per area was randomly chosen, consisting of 6 unique neurons and 6 shared neurons. Shared neurons simulated features characterizing all instances patterns of a category; they simulated shared conceptual features of all category members (category-critical feature, e.g., members of the first category are robots in the same height and are equipped with one camera, one speaker, two antennae, a power button, two metal legs, and a pair of shoes; members of the second category are cats and have round-shaped head, eyes, nose, mouth, ears, and whiskers; Fig. 1*B*, left). Unique neurons simulated the “idiosyncratic”, fully instance-specific visuomotor features; each of the corresponding feature neurons was only available in one instance pattern (e.g., robots vary in the body shape and color, the orientation of antennas, leg forms, the position of the power button, and shoe color). In sum, each category possessed 36 unique neurons from its 6 exemplars and 6 shared neurons. For word form patterns, category term patterns (ii) of within-category instances consisted of the same twelve neurons, which were coactivated with each of the three learnt grounding patterns of a category (e.g., to simulate the artificial words *fos* for all instances of the robot category, and *coxt* for all instances of the cat category; Fig. 1*B*, middle); each proper name pattern (iii) comprised twelve neurons, which were coactivated with one specific grounding pattern (e.g., *xub*, *vit*, and *hek* for the three instances of the robot category, respectively; Fig. 1*B*, right). The choice of cells for pattern generation was pseudorandomized and constrained by the following criteria. First, within-category neurons had to be nonadjacent to each other. This prevented coactivation merely due to close distance. Second, no grounding patterns from two different categories shared any neuron. Last, for each instance, the grounding patterns in *V1 and *M1_L_ followed the same principles but were not identical. The same rules applied to the grounding patterns in *A1 and *M1_i_.

### Experimental design

The current simulations involved three phases, model initialization, training phase, and testing phase, which were carried out on the high-performance computing system of Freie Universität Berlin (Bennett et al., 2020). During training, there were three different stimulation conditions, (1) where grounding patterns were learnt without symbol (no-symbol or control condition), (2) where all grounding patterns of each category were presented together with the same word form pattern (category term condition), and (3) where each grounding pattern was copresented with its own specific word form pattern (proper name condition). Thus, during learning, a stimulation pattern included two activation patterns (to *V1 and *PF_L_) when it was learned outside symbol context (Fig. 1*C*, top) or a quadruplet including the two instance-related patterns plus two-word form-related ones (to A1 and PF_i_) when learned in symbol context (Fig. 1*C*, bottom). Each test trial began with the presentation of a grounding pattern of an instance (projected to the two sensorimotor model areas V1 and M1_L_).

#### Model initialization

One crucial step prior to training was model initialization, which randomized all synaptic links (and their corresponding weights) between within-area cells and between cells from connected areas. Twelve sets of such synaptic links and weights (i.e., 12 different instantiations of the randomly initialized neural network) were chosen, each set was then triplicated (cf. Schomers et al., 2017), and each of these three copies entered one of the three training conditions—either no symbol, category term, or proper name. The use of distinct model instantiations can be seen as analogous to a within-subject study design with 12 subjects. We chose to implement three separate sets of simulations for the three conditions to avoid any possible interference effects between concepts and symbols that may emerge during training. Note, for example, that the relatively large representations that formed for CT might have interfered with further learning or may even have suppressed the activation of conceptual representations without symbols. This configuration yielded a controlled “within-subject” design with the training condition being a three-level repeated measure factor (*no symbol*, *category term*, and *proper name*). For the additional simulations performed to balance the number of word form presentations, there were four levels.

#### Training phase

The neural network model was repeatedly presented with 30 instances from ten categories. To mimic visuomotor percepts associated with an instance, the extrasylvian primary sensorimotor areas, *V1 and *M1_L_, were each presented with their grounding pattern (i) for 16 time steps. Following the experiment by LaTourrette and Waxman (2020) where instances were called either by a consistent label or by distinct labels each, our within-category trained instances were either paired with the same category term, by their distinct PN, or they were not labeled at all. To mimic symbols in the category term and proper name conditions, we presented to the primary perisylvian areas *A1 and *M1_i_ word form pattern (ii and iii), respectively, for 16 time steps (Fig. 1*C*, bottom, 2*A*). Hence, in different “learning trials,” the word form patterns of CT were copresented with one of three different grounding patterns from one category, whereas those of PN co-occurred with only one specific grounding pattern. There were no word form patterns presented in the baseline no-symbol condition to control for the effect of either type of linguistic label compared with learning without one (Fig. 1*C*, top, 2*A*).

Because activity at the end of a trial might affect learning in the next trial, the network was allowed to deactivate after each stimulated learning trial. To this end, we separated every two consecutive pattern stimulations by a waiting interval during which only the uncorrelated white noise mimicking spontaneous baseline neuronal firing was supplied to all areas (see Principle 6 in Model description—Neurobiological constraints). The goal was to reset the global network (i.e., all excitatory and inhibitory cells displayed a membrane potential of zero) before a new grounding pattern was inputted into the neural network model. This interstimulus interval was terminated only after the network activity had returned to its baseline value (thresh = 0.18, Table 2). As a result, the training order was not influential in this experiment.

**Table 2.** Parameter values used in the simulations

Equation 1	Time constant (excitatory cells)	τ=2.5 (time steps)
	Time constant (inhibitory cells)	τ=5 (time steps)
	Total input rescaling factor	$k_{1}=0.01$
	Noise amplitude	$k_{2}=7\sqrt{\left(24/\Delta t\right)}$ (Δt=0.5ms)
	Global inhibition strength	$k_{G}=0.80$ (time steps)
Equation 3	Spiking threshold	thresh=0.18
	Adaptation strength	α=8.0
Equation 4	Adaption time constant	$\tau_{\mathrm{ADAPT}}=10$ (time steps)
Equation 5	Rate estimate time constant	$\tau_{\mathrm{Favg}}=30$ (time steps, training)
		$\tau_{\mathrm{Favg}}=5$ (time steps, testing)
Equation 6	Global inhibition time constant	$\tau_{\mathrm{FGLOB}}=12$ (time steps)
Equation 7	Postsynaptic potential thresholds	$\vartheta_{+}=0.15$ (LTP)
		$\vartheta_{-}=0.14$ (LTD)
	Presynaptic output activity required for any synaptic change	$\vartheta_{\;pre}=0.05$ (LTP)
	Learning rate	Δw=0.0008

For details and a more elaborate discussion of the corresponding equations as well as their mathematical implementations, please see Henningsen-Schomers et al. (2022).

To balance learning conditions (NoS, CT, PN), each experiential grounding pattern representing an instance was presented 2,000 times in one set of simulations. However, because each category term pattern was copresented with three different instance patterns, whereas proper name patterns co-occurred with only one, this design leads to an imbalance of the number of learning trials during which individual word form patterns were presented (three times higher for category term than for proper name presentations; Fig. 2*C*, top). Therefore, a second evaluation of learning trials was performed and analyzed for which the number of word form pattern activations was balanced. In this case, there were 1,000 learning trials in the category term condition (CL_1x; each instance was presented together with a category term in 1,000 training trials, resulting in a total of 3,000 training trials per CT) and 3,000 trials in the proper name condition (PN_3x; each instance was presented together with a proper name in 3,000 training trials, resulting in a total of 3,000 training trials per proper name). For the control no-symbol conditions, two comparison values were calculated, after 1,000 (NoS_1x) and 3,000 (NoS_3x) trials (i.e., each instance was presented without symbol in 1,000 and 3,000 training trials, respectively; Fig. 2*C*, bottom). These different subdesigns are summarized graphically in Figure 2*C*.

#### Testing phase

In the current experiment, we implemented a version of an old-new recognition task with the use of new instances. For each of the ten categories, we presented to the neural network six testing instances: three trained instances and three novel instances (Fig. 2*B*). In total, we used 30 previously learnt instances and 30 new instances. However, no actual old-new pairing took place because we presented trained and novel instances to the neural network in separate test trials.

Memory performance of the network model was assessed in the absence of linguistic cues, i.e., without stimulating the perisylvian primary areas *A1 or *M1i. To stimulate the experience of individual instances, the extrasylvian primary areas *V1 and *M1_L_ were activated for two time steps with pure (i.e., free of any white noise) grounding patterns (i) and subsequentially deactivated toward the baseline for 28 time steps. We recorded network responses 30 time steps from the onset of this stimulation. Global resetting between two consecutive trials was conducted in the same manner as the training phase. Hence, the test order was not of interest.

### Data analysis

Grounding pattern production, data processing, and data analysis were performed using Python 3.9.7, matplotlib 3.4.3 (Hunter, 2007), NumPy 1.20.3 (Harris et al., 2020), pandas 1.3.4 (Reback et al., 2022), SciPy 1.7.1 (Virtanen et al., 2020), and seaborn 0.11.2 (Waskom, 2021). In the current work, statistical significances were based on a conservative *p* value threshold of 0.005 suggested by Di Leo and Sardanelli (2020). We used rstatix 0.7.0 (Kassambara, 2021) in the R software environment (R Core Team, 2021) for statistical analyses.

When testing stimuli were presented to the primary sensorimotor areas, some of the 625 excitatory neurons per area fired in response to their conceptual grounding patterns. As described in the procedure, we recorded all their responses during 30 time steps from stimulation. Let ϕ(et) denote the output of an excitatory cell e at time t, such that ϕ only takes up the value 0 or 1 and t only allows discrete values up to 30 (corresponding to thirty possible simulation time steps); let $\tau_{\mathrm{Favg}}=5$ be a time constant, and the estimated instantaneous firing rate $\omega_{E}(e\;\;t)$ of cell e at time t can be calculated based on the following equation:

$$\tau_{\mathrm{Favg}}\cdot \frac{d\omega_{E}(e\;\;t)}{dt}=-\omega_{E}(e\;\;t)+\phi (e\;\;t)$$ (1)

Solving Equation 1 for $\omega_{E}(e\;\;t)$ returns the cell's latest spiking activity (firing rate). We estimated the mean firing rate based on $t=t_{30}$ and used this value for the subsequent RSAs. For details about relevant calculation steps, see the Appendix in the study of Henningsen-Schomers and Pulvermüller (2022).

Previous research found that several of the extrasylvian areas targeted by the deep neural model (including, for example, *V1 and *AT) are important for processing instance- and concept-related information (Binder et al., 2005; Martin, 2007; Ralph et al., 2017; Henningsen-Schomers et al., 2022). Therefore, the current data analyses and statistical testing focused on the extrasylvian region of the deep neural network. This decision was motivated by the main aim of addressing possible causal influences of symbol learning on the perceptual processing of instances of concepts and on conceptual processing itself.

#### RSA

**Figure 3.**
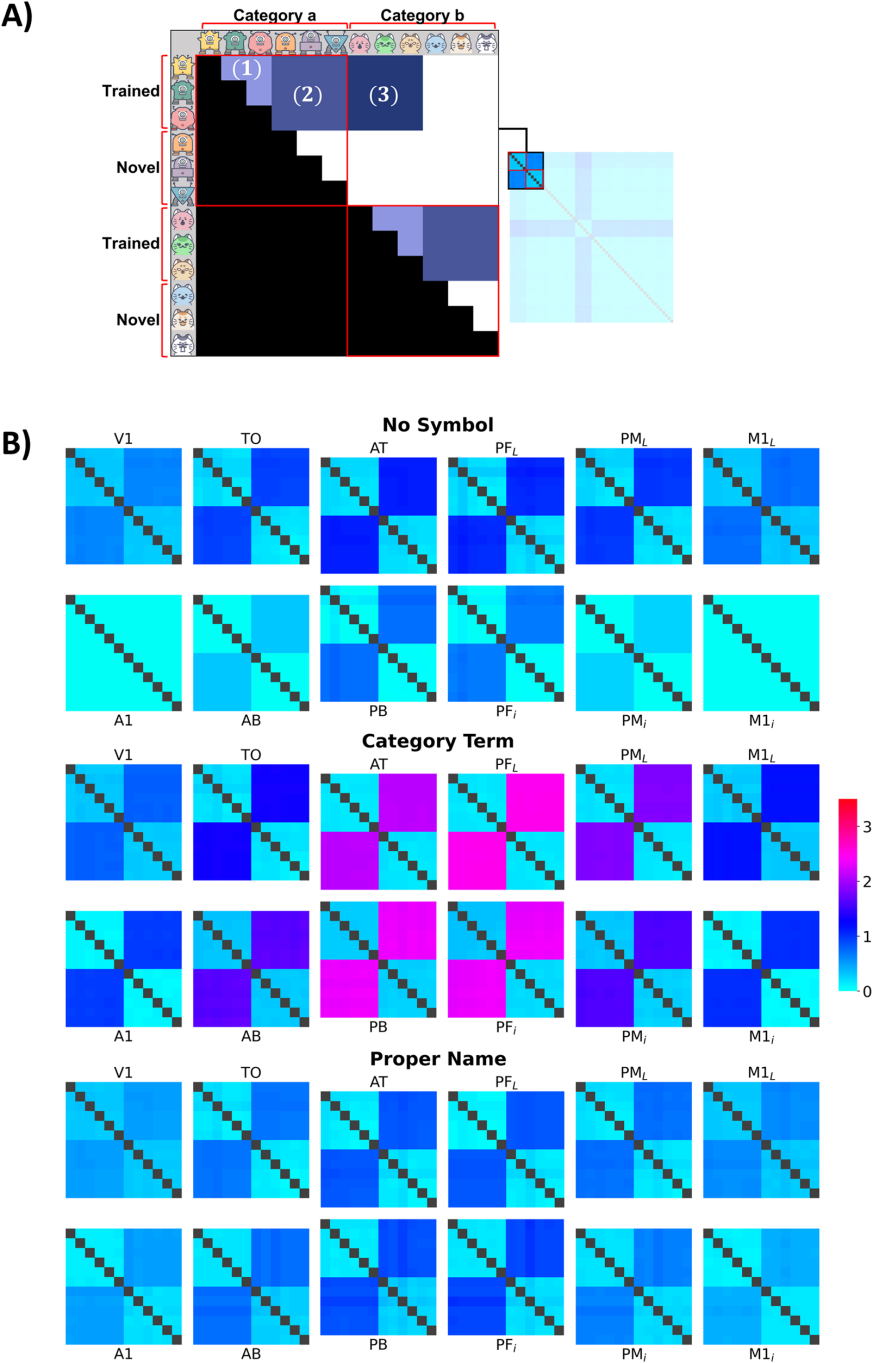
***A***, Schematic extraction of a 60×60 RDM, which represents 12 instances from two different categories and the similarities between any instance pair. For illustration, we once again use the categories of robots and cat faces. The schematic dissimilarity matrix illustrates how between-category (cells outside the red boundaries) and within-category dissimilarities (cells within the red boundaries) were calculated. Of interest are the (1) within-category dissimilarity among trained instances ( $\mathrm{Dissi}m_{W-TT}$, lightest blue shade), (2) within-category dissimilarity between a trained and a novel instance ( $\mathrm{Dissi}m_{W-TN}$, intermediate blue shade), and (3) between-category dissimilarity of two trained instances ( $\mathrm{Dissi}m_{B-TT}$, darkest blue shade). The RDM is symmetric about its diagonal (gray) of zeros (representing the nondissimilarity of each of the instances to itself). Only the upper half of the RDM is used for analysis, and the lower half could be abandoned (black). ***B***, RDMs for each of the twelve model areas in three main simulations: no symbol (top row), category term (middle row), and proper name (bottom row). The squares indicate the degrees to which network activity in the 12 network areas elicited by (12 out of 60) grounding patterns in the three learning conditions differed between each other within and between categories and are color-coded from turquoise (no dissimilarity, Dissim=0), blue, pink, and to dark red (high dissimilarity, Dissim>3).

The estimated mean firing rate of 625 neurons in response to a testing instance reflected how this instance was represented in a neural network. To understand how differently the neural network represented within- and between-category instances, we calculated the dissimilarity in firing patterns for every pair of the 60 instances. Pairwise dissimilarities computed in terms of Euclidean distance were organized in a 60×60 representational dissimilarity matrix (RDM; Fig. 3*A*). Each cell in the matrix reflected the dissimilarity between the firing patterns of two instances. In total, there were 36 RDMs across 3 training conditions and 12 areas.

We defined two classes of pairwise dissimilarities, including between-category dissimilarity $\left(\mathrm{Dissi}m_{B}\right)$ and within-category dissimilarity $\left(\mathrm{Dissi}m_{W}\right)$. A second way to define similarity types is based on the type of instances under study, that is, the dissimilarity between two trained instances $\left(\mathrm{Dissi}m_{TT}\right)$, between two novel instances $\left(\mathrm{Dissi}m_{NN}\right)$, and between a trained and a novel instance $\left(\mathrm{Dissi}m_{TN}\right)$. For example, within-category dissimilarity could be classified as either dissimilarity among trained instances 1–3 $\left(\mathrm{Dissi}m_{W-TT}\right)$, among novel instances 4–6 $\left(\mathrm{Dissi}m_{W-NN}\right)$, or between trained and novel instances $\left(\mathrm{Dissi}m_{W-TN}\right)$ (Fig. 3*A*).

##### Category learning

Category learning was evaluated through the ability to (1) distinguish differences between categories and (2) group together category members. We assessed how different types of symbols impacted upon category learning performance based on (1) the dissimilarity between two between-category trained instances $\left(\mathrm{Dissi}m_{B-TT}\right)$ and (2) the dissimilarity between two within-category trained instances $\left(\mathrm{Dissi}m_{W-TT}\right)$ (Fig. 3*A*). Successful category learning occurred when two instances from two distinct categories were considered as dissimilar (high $\mathrm{Dissi}m_{B-TT}$) and/or when two within-category instances were considered as similar (low $\mathrm{Dissi}m_{W-TT}$). If, as previously claimed, applying CT invites one to encode the commonalities among instances and thereby facilitates categorization, the deep neural network should represent within-category instances similarly while highlighting the dissimilarities between instances of different categories. In the category term condition, we expected between-category dissimilarities to be greater than within-category dissimilarities $\mathrm{Dissi}m_{B-TT_{CT}}>\mathrm{Dissi}m_{W-TT_{CT}}$. In contrast, we proposed two scenarios for the proper name condition. In the first scenario, if PN focus the neural network models on encoding only unique features and inhibit the encoding of category-critical features, no traces of category learning will be observable, and the representations of individual instances will be highly dissimilar regardless of their categorical membership $\left(\mathrm{Dissi}m_{B-TT_{PN}}\approx \mathrm{Dissi}m_{W-TT_{PN}}\right)$. However, because within-category instances shared 50% of their activated neurons in the extrasylvian primary areas *V1 and *M1_L_, the neural network could base on such similarities to form category representation. In this second scenario, PN are not sufficient to override category learning; the neural network would house not only the unique representations of the instances but also the commonalities of those belonging to the same category. Like the category term condition, the test data would also show signs of category learning $\left(\mathrm{Dissi}m_{B-TT_{PN}}>\mathrm{Dissi}m_{W-TT_{PN}}\right)$. Taking into account such intrinsic perceptuomotor similarities among instances from the same category, category learning was evaluated not only across symbol (i.e., category term or proper name) learning conditions but also in control conditions (i.e., training without symbols). For example, a superior causal influence of CT on category learning performance would be expressed through a significantly higher $\mathrm{Dissi}m_{B-TT_{CT}}$ and lower $\mathrm{Dissi}m_{W-TT_{CT}}$ relative to training with PN and also relative to training without symbols.

##### Generalization

Assuming the neural network had encoded the commonalities between within-category trained instances and formed category knowledge with the help of these shared features, they might have as well represented novel instances as members of that category when exposed to the category-critical features in these novel instances. Generalization performance would then be reflected by how similarly within-category trained instances and within-category novel instances stimulated the deep neural network. To evaluate the generalization performance of the neural network on novel instances, pairwise dissimilarities between two trained instances $\left(\mathrm{Dissi}m_{W-TT}\right)$ as well as between a trained and a novel instance $\left(\mathrm{Dissi}m_{W-TN}\right)$ were extracted. In the testing phase, the chance was low that the neural network readily applied category knowledge earned from thousands of training trials onto a novel instance in the first and only exposure. In the case of poor generalization performance, the activation pattern of within-category novel instances would be dissimilar from that of the within-category trained instances (i.e., increasing $\mathrm{Dissi}m_{W-TN}$). Our criterion for a successful generalization after learning with symbols was that $\mathrm{Dissi}m_{W-TN}$ should be as low as $\mathrm{Dissi}m_{W-TT}$ $\left(\mathrm{Dissi}m_{W-TN}\approx \mathrm{Dissi}m_{W-TT}\right)$. In other words, their absolute dissimilarity difference $\mathrm{DissimDiff}=|\mathrm{Dissi}m_{W-TN}-\mathrm{Dissi}m_{W-TT}|$ must remain lower than when the deep neural network was trained without symbols.

#### Cell assembly analysis

Motivated by the notion of cell assemblies (CAs; Hebb, 1949; Braitenberg, 1978; Fuster, 2005), that is, strongly interlinked sets of neurons forming as a consequence of correlated neuronal activity and potentially carrying a main role in cognitive brain processing, we conducted cell assembly analyses to discover possible neuronal correlates of grounding instances, concepts and symbols along with instance-specific and category-critical neurons after repeated exposure to instances and their CT or PN. We extracted CAs activated by each of the 60 grounding patterns used as testing instances based on the criterion described in previous work (Garagnani and Pulvermüller, 2016; Henningsen-Schomers and Pulvermüller, 2022). Grounding patterns in the testing phase tended to coactivate several excitatory neurons (e-cells) in an area, with at least one being maximally responsive (nonresponse was under the threshold of 0.01). To be part of a CA, the firing rate of a given e-cell had to exceed 75% of the firing rate of the maximally responsive cell of the same area. We then computed the number of unique, instance-specific and overlapping, and conceptual neurons among CAs for trained instances of the same category: neurons were classified according to whether they were activated by just one grounding pattern or whether they responded to two or three instances (thus being pair or triple-shared between the learnt instances of a concept). Unique neurons were conceptualized as neurons that encoded specific, “idiosyncratic” features of an instance; shared neurons could be understood as those that encoded common features shared by at least two instances and thus characteristic of their category. The specialized encoding of category-critical features could be indicated by a higher proportion of shared neurons per area, while traces of instance-specific features would be reflected by a larger proportion of unique neurons.

Representations are transformed through different levels of processing, i.e., from the primary areas to secondary areas, and the central “connector hub” areas of the model. We quantified such transformation as the change (i.e., gain/loss) in the number of unique and shared CA cells in the extrasylvian central areas (AT, PF_L_) comparative to the extrasylvian primary areas (V1, M1_L_). Gains in a type of neuron, for example, shared neurons, are indicative of intensive encoding of concept-related commonalities on the course of processing, while loss of shared neurons in the central areas implies reduced encoding of idiosyncratic features and hence instance-related information. Percentage gain was calculated as the difference between the number of neurons in the central and primary areas, as a percentage with respect to the number of neurons in the primary areas:

$$\mathrm{Gain}=\frac{n_{\mathrm{central}}-n_{\mathrm{primary}}}{n_{\mathrm{primary}}}\times 100$$

##### Representations of category-critical features

A range of previous neurocomputational studies show that, when brain-like networks learn concepts and word meanings, they form CAs that are spread out across sensorimotor and more central areas of the network. The density of shared semantic neurons in the most central connector hubs is greatest due to their high connectivity degree and thus ample convergence of activity in these areas, resulting in especially strong activation, in particular for shared semantic neurons (for discussion, see Garagnani et al., 2017; Tomasello et al., 2018). Relative to instance-specific neurons, shared semantic neurons are activated more frequently during semantic learning, which predicts that these will recruit the largest number of additional cell assembly; these would therefore be semantic, too, and primarily located in the central hub regions. If a labeling condition specifically invites the neural network to encode category-relevant features, we expect (1) more shared neurons than unique neurons in the extrasylvian areas and (2) a greater gain in shared neurons in the central semantic areas compared with the primary areas. Category learning might still occur even in the presence of PN because within-category similarities also characterize sensorimotor experiences. If such information is sufficient, there should be traces of shared neurons in the central, multimodal areas as well. Additionally, CT should activate shared neurons more than PN.

##### Representations of instance-specific features

When a neural network represents instances as unique entities, it shall reveal specific traces of each instance in the extrasylvian areas, especially in the semantic hubs. In an extreme case where category learning is hindered and the neural network only encodes the uniqueness of instances, there should be (1) more unique than shared neurons in the extrasylvian areas and (2) a gain only in unique neurons in the central areas with respect to the primary areas. Importantly, instances with PN are expected to activate significantly more unique neurons than categorically labeled instances.

We gather from all 12 model instantiations the CAs in response to all 30 trained instances of 10 categories and classify CA cells by their uniqueness to each instance (vs sharedness). To facilitate readers’ understanding about the results, we offer an interactive illustration of these CAs on our web application at (https://phucthuun.shinyapps.io/CL_PN/). This web application enables one to compare the differential effects of CT versus PN in representing category-critical and instance-specific features of within-category and across-category instances.

## Results

### RSA

Figure 3*B* gives a first impression of the instance and category learning performance after 2,000 training trials. In the category term condition, instances from the same category activated the neural network similarly, whereas instances from different categories led to substantially more dissimilar activation patterns across the different areas of the network (i.e., firing patterns were highly dissimilar, as color-coded by dark blue and pink). Category knowledge was reflected in a relatively reduced dissimilarity (light blues), which appears as homogenous within each category, contrasting with those between categories, especially in the central areas (semantic hubs). Training the deep neural network without the aid of symbols or with PN reduced the networks’ ability to distinguish instances between categories: activity pattern dissimilarities between instances from different categories were much more substantial in the category term condition than in the proper name condition (color-coded with shades of intermediate blue). In contrast, within-category similarities and generalization performance in the category term condition were superior, as indicated by the more homogeneous (light) blue shade across all six instances (trained and not trained) from the same category, relative to the other two conditions, where different shades of light blue are visible.

#### Category learning

**Figure 4.**
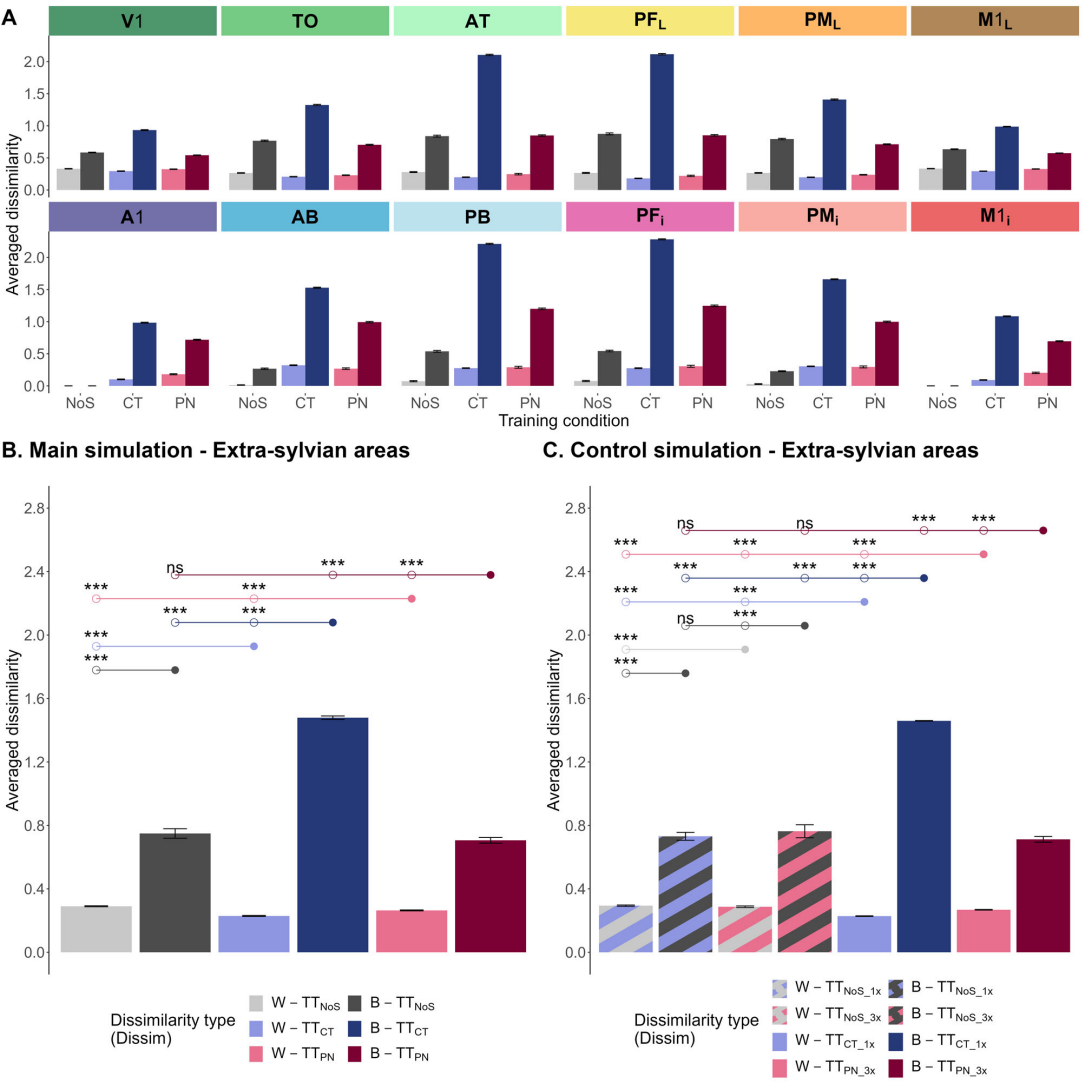
Bar charts depicting dissimilarities between network activity elicited by trained grounding patterns after learning for each of the three training conditions. ***A***, Main simulation: within-category (W-TT) and between-category (B-TT) dissimilarity values across all 30 trained activity patterns were averaged for each of the twelve model areas. ***B,C***, Within-category (W-TT) and between-category (B-TT) dissimilarities across the 30 trained items were averaged for extrasylvian model areas. The three training conditions of the main simulations (***B***) were no symbol (NoS, gray), category term (CT, blue), and proper name (PN, pink). The four training conditions of the control simulation (***C***) were no symbol with each instance presented over 1,000 (NoS_1x, blue-striped gray) or 3,000 trials (NoS_3x, pink-striped gray), Category term where each instance presented over 1,000 trials (CT_1x, blue) and proper name where each instance presented over 3,000 trials (PN_3x, pink). The error bars represent 95% confidence intervals of the mean. The circles above the bars represent post hoc pairwise comparisons between a reference (circles with filled colors) and a corresponding mean (unfilled circles) after Bonferroni’s correction (critical *p* value = 0.005). Ten comparisons relevant to the main effects of training condition and dissimilarity type and their interaction are illustrated. The asterisks represent two-tailed *p* values: **p < 0.005, and ***p < 0.001, ns, not significant. The results were replicated in the whole model architecture (six extrasylvian and six perisylvian model areas); see Extended Data Figure 4-1 and Extended Data Table 4-1.

To evaluate category learning performance after 2,000 learning trials, within-category dissimilarity $\left(\mathrm{Dissi}m_{W-TT}\right)$ and between-category dissimilarity between activity patterns elicited by grounding patterns of trained instances $\left(\mathrm{Dissi}m_{B-TT}\right)$ were used. Figure 4*A* describes a global tendency of the deep neural network, across its twelve areas and three training conditions, to identify within-category instances as more similar and between-category instances as more dissimilar to each other. This feature is explained by the grounding patterns presented, which were similar across category instances, but not between. However, between-category dissimilarity is relatively enhanced in central areas, a feature not explained by the stimulations. In the next step, dissimilarity values were averaged for the six extrasylvian areas. The two-factorial repeated measure (3×2) – ANOVA with training condition (no symbol/category term/proper name) and dissimilarity type $\left(\mathrm{Dissi}m_{W-TT}/\mathrm{Dissi}m_{B-TT}\right)$ confirmed the main effect of both factors ( $F_{\left(2\;22\right)}=2777.647$, $p<0.001\;\;\;\;\eta^{2}=0.982$ and $F_{\left(1\;11\right)}=11155.611\;\;\;\;p<0.001\;\;\;\;\eta^{2}=0.996$, respectively) as well as their interaction effect ( $F_{\left(2\;22\right)}=6113.987$, $p<0.001\;\;\;\;\eta^{2}=0.986$) on the dissimilarity between instances within these extrasylvian areas. Figure 4*B* illustrates category-related activation performance of the deep neural network in the extrasylvian areas of the three learning conditions: the neural network successfully grouped together instances from the same category while distinguishing between instances from the same versus from two different categories. Pairwise comparisons with Bonferroni’s correction were computed to observe the effect of training conditions on each level of dissimilarity type and vice versa. The results showed that $\mathrm{Dissi}m_{W-TT}$ was significantly lower than $\mathrm{Dissi}m_{B-TT}$ in all three conditions (ps<0.001); same category membership was thus manifest as relatively enhanced activation similarity in all conditions and across areas. The $\left(\mathrm{Dissi}m_{W-TT}\right)$ in the category term condition (*M* = 0.229, SD = 0.005) and the proper name condition (*M* = 0.264, SD = 0.004) was significantly smaller (i.e., greater similarity) than that in the control no-symbol condition (*M* = 0.29, SD = 0.006), and they were also significantly different from each other, with greatest similarities after category term labeling (ps<0.001). Relative to the control no-symbol condition, the deep neural network responded similarly to trained instances coming from the same category when it was trained with symbols and such performance was above baseline. Importantly, the benefit of CT was superior to both training without symbols and with PN. Likewise, the deep neural network returned the highest $\mathrm{Dissi}m_{B-TT}$ (*M* = 1.48, SD = 0.018) for the category term condition (ps<0.001), while $\mathrm{Dissi}m_{B-TT}$ in the proper name condition (*M* = 0.706, SD = 0.01) was not significantly different from that in the no-symbol condition (*M* = 0.749, SD = 0.045) (p=0.01), after application of the Bonferroni’s-corrected significance threshold of 0.005. Compared to the no-symbol condition, training with PN only gradually hindered the discrimination of between-category instances but left the separation of within-category instances unaffected. In contrast, both aspects of category learning were present with the aid of CT, reduced within- and enhanced between-category similarities.

10.1523/JNEUROSCI.1048-23.2023.f4-1 Figure 4-1 Bar charts depicting dissimilarities between network activity elicited by trained grounding patterns after learning for each of the three training conditions. Within-category (W-TT) and between-category (B-TT) dissimilarities across the 30 trained items were averaged for **A-B)** all 12 model areas and **C-D)** extrasylvian model areas. For further explanation, see Figure 4. Download Figure 4-1, TIF file.

10.1523/JNEUROSCI.1048-23.2023.t4-1 Table 4-1 ANOVA table reporting significant effects of training condition (No symbol/Category term/Proper name) and dissimilarity type $\left(Dissim_{W-TT}/Dissim_{B-TT}\right)$ on averaged dissimilarity across 12 model areas. Download Table 4-1, DOCX file.

The simulations performed to control for the number of word form presentations during learning were evaluated using a two-factorial repeated measure (4×2) ANOVA with training condition (now four levels, NoS_1x/NoS_3x/CT_1x/PN_3x) and dissimilarity type $\left(\mathrm{Dissi}m_{W-TT}/\mathrm{Dissi}m_{B-TT}\right)$. This confirmed the main effect of both factors ( $F_{\left(1.67\;18.35\right)}=1113.758$, p<0.001, $\eta^{2}=0.964$ and $F_{\left(1\;11\right)}=7485.295$, p<0.001, $\eta^{2}=0.993$, respectively) as well as their interaction effect $(F_{\left(1.65\;18.10\right)}=1961.497$, $p<0.001\;\;\;\;\eta^{2}=0.973)$ on the dissimilarity between instances within extrasylvian areas. Pairwise comparisons with Bonferroni’s correction were computed to observe the effect of training conditions on each level of dissimilarity type and vice versa. In essence, $\mathrm{Dissi}m_{B-TT}$ in the category term condition was significantly higher than that in the proper name and both no-symbol control conditions (ps<0.001) (Fig. 4*C*); category term learning increased the dissimilarity across conceptual categories relative to no-symbol learning and proper name learning. The reverse effect, greater dissimilarity values for PN than CT, was found within categories. These observations were therefore valid even when PN were “shown” to the model three times more than CT during learning.

#### Generalization

**Figure 5.**
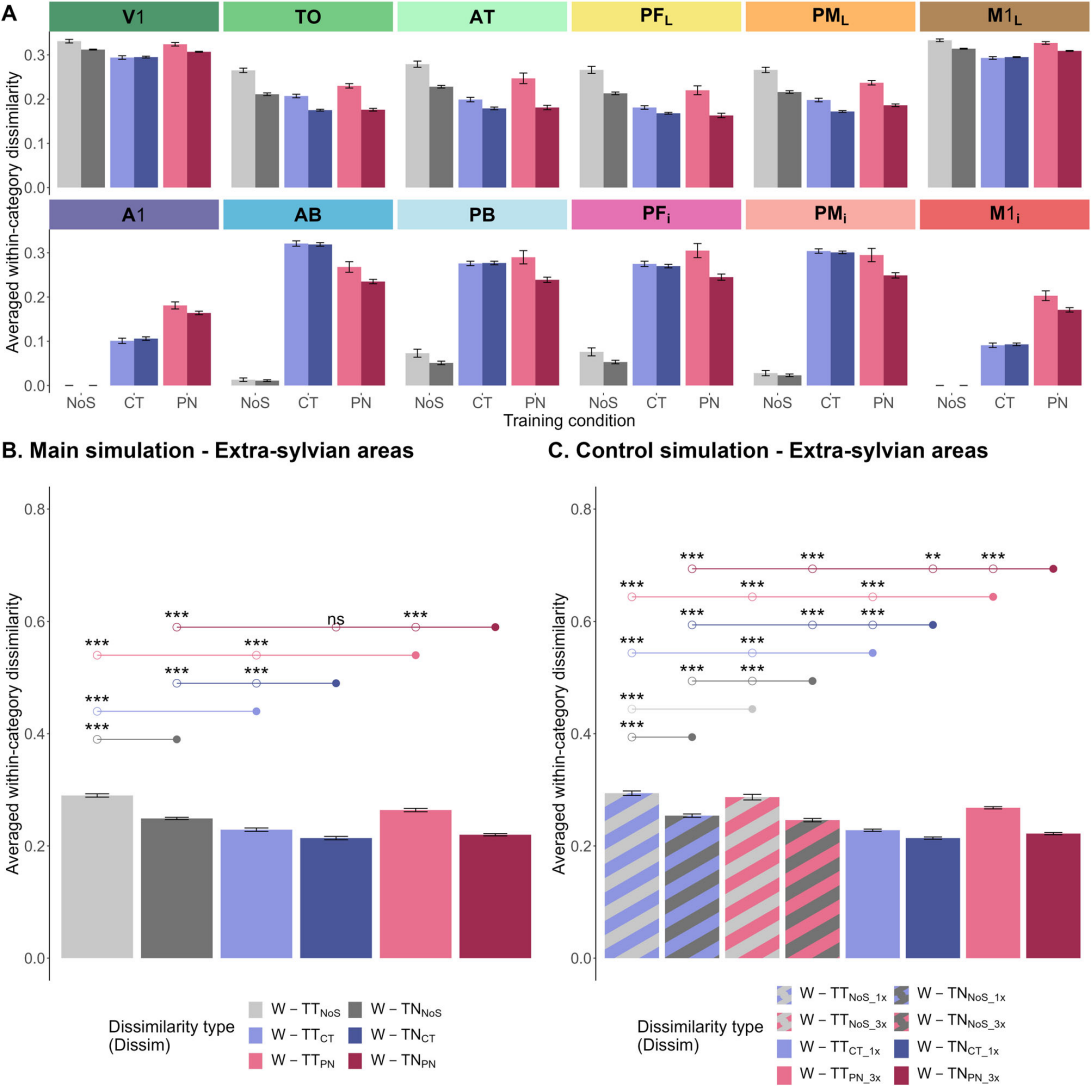
Bar charts depicting dissimilarities between network activity elicited by trained novel grounding patterns after learning for each of the three training conditions. ***A***, Main simulation: within-category dissimilarity values between any two trained instances (W-TT) and between trained and novel instances were averaged for each of the twelve model areas. ***B***,***C*** Within-category dissimilarities between any two trained instances (W-TT) and between trained and novel instances (W-TN) were averaged for extrasylvian model areas. The three training conditions of the main simulations (***B***) were no symbol (NoS, gray), category term (CT, blue), and proper name (PN, pink). The four training conditions of the control simulation (***C***) were NoS_1x (blue-striped gray) or NoS_3x (pink-striped gray), CT_1x (blue) and PN_3x (pink). For further explanation, see Figure 4. The results were replicated in the whole model architecture (six extrasylvian and six perisylvian model areas); see Extended Data Figure 5-1 and Extended Data Table 5-1.

To evaluate the generalization performance of the deep neural network on novel instances, pairwise dissimilarities between two trained instances $\left(\mathrm{Dissi}m_{W-TT}\right)$ as well as between a trained and a novel instance $\left(\mathrm{Dissi}m_{W-TN}\right)$ were used. Figure 5*A* illustrates the tendency of the deep neural network to represent two trained instances of the same category as more dissimilar, whereas the representations of a novel and a trained instance from the same category were less dissimilar (lighter-shaded columns were mostly higher than darker-shaded columns). In the six extrasylvian areas, a 3×2 ANOVA was computed with training condition (no symbol/category term/proper name) and type of within-category dissimilarity $\left(\mathrm{Dissi}m_{W-TT}/\mathrm{Dissi}m_{W-TN}\right)$ as repeated measure factors. Both the main effects of training condition $\left(F_{\left(2\;22\right)}=465.217\;\;\;\;p<0.001\;\;\;\;\eta^{2}=0.956\right)$ and dissimilarity type $\left(F_{\left(1\;11\right)}=7711.618\;\;\;\;p<0.001\;\;\;\;\eta^{2}=0.939\right)$ were significant. For these two factors, there was also a significant interaction $\left(F_{2\;22}=635.788\;\;\;\;p<0.001\;\;\;\;\eta^{2}=0.707\right)$ (Fig. 5*B*). The Greenhouse–Geisser sphericity correction to the violated sphericity assumption (p=0.024) for training conditions $\left(\;p[\mathrm{GG}]=2.38\times 10^{-11}\right)$ confirmed this result. Two-sided pairwise comparisons with Bonferroni’s correction showed that $\mathrm{Dissi}m_{W-TN}$ in the category term (M=0.214SD=0.004) and in the proper name conditions (M=0.220SD=0.003) were significantly lower than that in the control no-symbol condition (M=0.249SD=0.004) (ps<0.001), but they did not differ significantly from each other (p=0.01) (Fig. 5*B*). $\mathrm{Dissi}m_{W-TN}$ was significantly lower than $\mathrm{Dissi}m_{W-TT}$ in all three conditions (ps<0.001) (Fig. 5*B*), which means that within-category trained instances were represented as less similar to each other than when each of them was compared with a novel instance from the same category. In other words, trained instances resulted in neuronal response patterns that were more similar to those caused by novel instances than those caused by trained instances from the same category, a finding easily explained by the lack of learning of the idiosyncratic features of novel instances. A further set of pairwise comparisons using Bonferroni’s correction revealed that the absolute DissimDiff in the no-symbol condition (M=0.041, SD=0.016) was significantly higher than DissimDiff in the category term condition (M=0.016SD=0.012) (p<0.001) but not significantly different from that in the proper name condition (M=0.044SD=0.02) (p=0.009). In other words, category term learning resulted in the most similar processing of learnt and not-learnt instances and thus to the greatest degree of generalization.

10.1523/JNEUROSCI.1048-23.2023.f5-1 Figure 5-1 Bar charts depicting dissimilarities between network activity areas elicited by trained novel grounding patterns after learning for each of the three training conditions. Within-category dissimilarities between any two trained instances (W-TT) and between trained and novel instances (W-TN) were averaged for **A&B)** all 12 model areas and **C&D)** perisylvian model areas. For further explanation, see Figure 4. Download Figure 5-1, TIF file.

10.1523/JNEUROSCI.1048-23.2023.t5-1 Table 5-1 ANOVA table reporting significant effects of training condition (No symbol/Category term/Proper name) and dissimilarity type $\left(Dissim_{W-TT}/Dissim_{W-TN}\right)$ on averaged dissimilarity across 12 model areas. Download Table 5-1, DOCX file.

The results from the additional simulations controlling for the number of word form presentations during learning (i.e., four training conditions NoS_1x, NoS_3x, CT_1x, PN_3x, see Materials and Methods) also confirmed that generalization was maximal for novel members of categories for which category term had been learned (Fig. 5*C*). The mere exposure to instances or learning PN showed little generalization relative to category learning.

These results investigating brain-constrained neural network correlates of conceptual generalization sit well with well-known observations that language-learning children often generalize—or even overcategorize—CT to novel items. In case of overgeneralization of an item, subsequent learning may establish a novel category to which the item belongs. While our results offer a mechanistic perspective on generalization, a detailed simulation of overgeneralization and reclassification learning is left for future study.

### Cell assembly analysis

**Figure 6.**
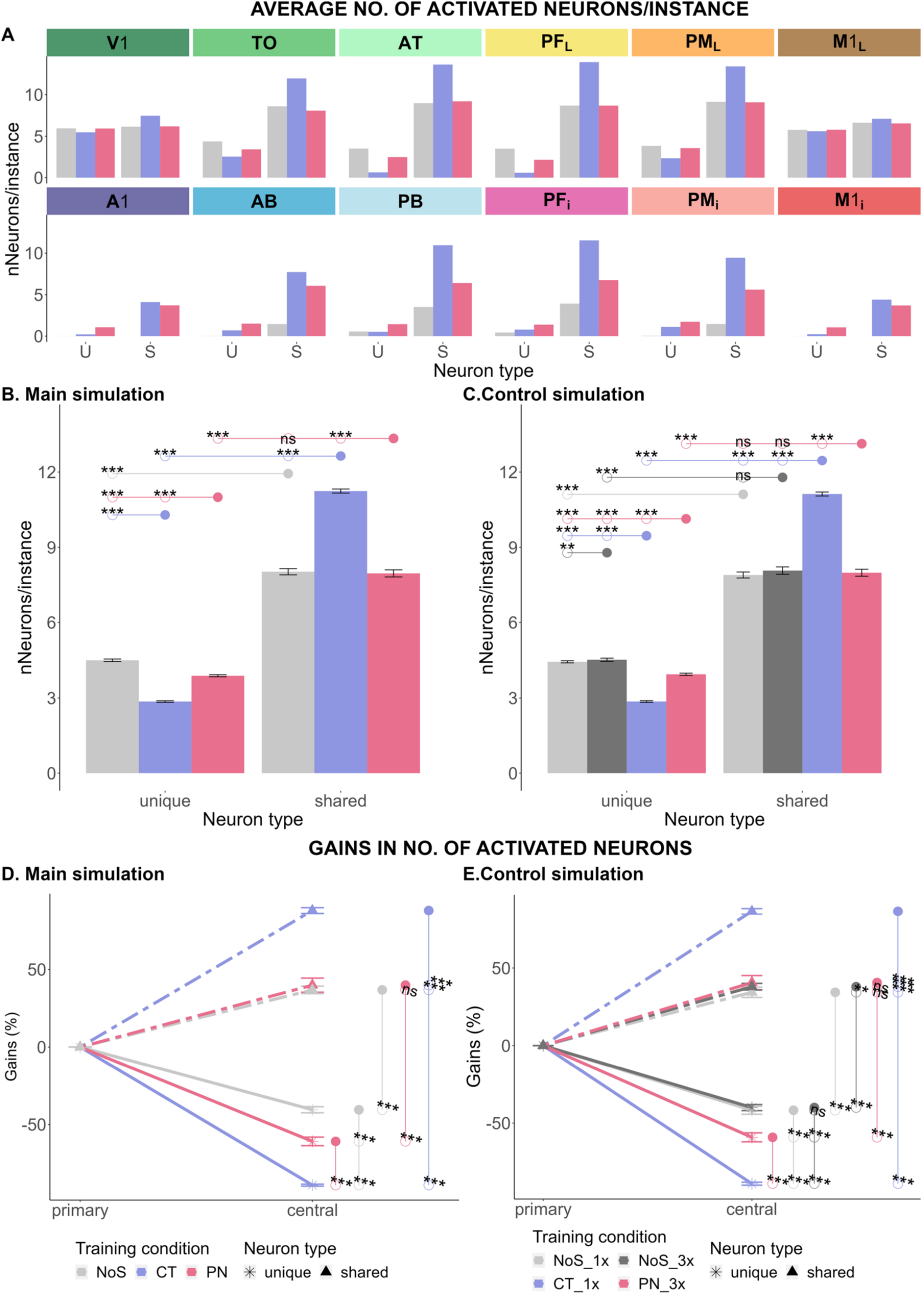
Bar charts depicting average numbers of instance-specific (“unique”) and category general (“shared”) neurons activated by grounding patterns of instances learnt in the three training conditions, no symbol (gray), category term (blue), and proper name (pink). ***A***, Main simulation: The number of activated unique (U) and shared (S) neurons in response to each of the 30 trained instances was averaged across all 12 model areas. ***B,C***, The number of activated neurons in response to the 30 trained grounding patterns was averaged for each of the six extrasylvian areas. ***D,E***, Changes in neuronal activation seen between extrasylvian primary areas, where stimulation was given, and the “higher” more central connector hub areas central to the architecture. Changes in the number of activated neurons in response to trained grounding patterns are shown for the three training conditions. Unique neurons are shown by solid lines with crossed ends and shared ones by broken lines with triangular ends. The three training conditions of the main simulations (***B,D***) were no symbol (NoS, gray), category term (CT, blue), and proper name (PN, pink). The four training conditions of the control simulation (***C***) were NoS_1x (blue-striped gray) or NoS_3x (pink-striped gray), CT_1x (blue) and PN_3x (pink). For further explanations, see Figure 4. The results were replicated in the whole model architecture (six extrasylvian and six perisylvian model areas); see Extended Data Figure 6-1 and Extended Data Table 6-1.

Figure 6*A* illustrates the tendency of the deep neural network to encode fewer unique neurons (U-shaped function across areas) and more shared neurons (inverted U-shaped function) in the extrasylvian central areas than in the extrasylvian primary areas. In the first step, the number of unique neurons and shared neurons activated by each instance were calculated and averaged across two training conditions. The repeated measure 3×2 ANOVA with training condition (no symbol/category term/proper name) and neuron type (unique/shared) confirmed the significant main effects ( $F_{\left(2\;22\right)}=902.098$, $p<0.001\;\;\;\eta^{2}=0.926$ and $F_{\left(1\;11\right)}=13966.410\;\;\;\;p<0.001\;\;\;\eta^{2}=0.998$, respectively) and a significant interaction involving both factors $\left(F_{2\;22}=5027.907\;\;\;\;p<0.001\;\;\;\eta^{2}=0.985\right)$. The supplementary 2×2 ANOVA with training condition with symbols (category term/proper name) and neuron type (unique/shared) returned comparable results with two significant main effects ( $F_{\left(1\;11\right)}=1009.255$, $p<0.001\;\;\;\eta^{2}=0.951$ and $F_{\left(1\;11\right)}=$ 23994.328, $p<0.001\;\;\;\eta^{2}=0.998$, respectively) and a significant interaction involving both factors $(F_{\left(1\;11\right)}=$ $4593.789\;\;\;\;p<0.001\;\;\;\eta^{2}=0.986)$. Pairwise comparisons with Bonferroni’s correction revealed that CT made the neural network reactivate more shared neurons (M=11.242, SD=0.127) than unique neurons (M=2.861SD=0.051) (p<0.001). This also applied for training with PN (shared neurons, M=7.963SD=0.222; unique neurons, M=3.89, SD=0.064) and training without symbols (shared neurons, M=8.029, SD=0.194; unique neurons, M=4.493, SD=0.08) (ps<0.001) (Fig. 6*B*). Compared to this control condition, the number of unique instance-specific neurons was moderately reduced by PN but radically so by CT (p<0.001), whereas the number of shared, conceptual category neurons remained unchanged after proper name learning (p=0.447) but increased dramatically with category term acquisition (p<0.001). The latter is clear evidence for a facilitatory effect of language, more specifically, of category term learning, on conceptual category formation in brain-constrained deep neural networks.

10.1523/JNEUROSCI.1048-23.2023.f6-1 Figure 6-1 **A &B)** The number of activated neurons in response to the 30 trained grounding patterns was averaged for each of the 12 model areas. **D&E)** Changes in neuronal activation seen between primary areas, where stimulation was given, and the ‘higher’ more central connector hub areas central to the architecturssssse. For further explanations see Figure 4. Download Figure 6-1, TIF file.

10.1523/JNEUROSCI.1048-23.2023.t6-1 Table 6-1 ANOVA table reporting significant effects (across 12 area model) of training condition (No symbol/Category term/Proper name) and neuron type (shared/unique) on the number of activated neurons (left) and on the gain/loss of unique/shared neurons from primary areas to the connector hub (right). Download Table 6-1, DOCX file.

With respect to the gain/loss of neurons in the extrasylvian central areas relative to the primary ones, our repeated-measure 3×2 ANOVA with two factors training condition (no symbol/category term/proper name) and neuron type (unique/shared) confirmed both main effects on the percentage change of neurons and their interaction to be significant ( $F_{2\;22}=55.17837$, p<0.001, $\eta^{2}=0.5519424$, $F_{\left(1\;11\right)}=6471.54090$, p<0.001, $\eta^{2}=0.9954$, and $F_{\left(2\;22\right)}=1484.43893$, p<0.001, $\eta^{2}=0.966$, respectively). According to the subsequent pairwise *t* tests, the deep neural networks gained shared neurons but lost unique neurons in the central areas, which held true for all conditions (ps<0.001) (Fig. 6*D*, upward dotted lines represent positive gains in shared neurons and downward solid lines mean negative gains in unique neurons). On the three levels of training condition, the gain in shared neurons and the loss in unique neurons in the category term condition were significantly larger than that in the proper name and no-symbol conditions (ps<0.001) (Fig. 6*D*). PN did not significantly increase the gain in shared neurons (p=0.1) but led only to a moderate loss of unique neurons, as compared with the control training condition (ps<0.001). These results further confirm that training with CT magnified both the gain in shared semantic neurons in central areas and the loss of unique instance-specific neurons there. The simulations performed for balancing the number of word form presentations during proper name and category term learning also confirmed these observations (Fig. 6*C,E*). Therefore, the overgrowth of shared neurons in category term learning does not depend on an abundant number of word form presentations and cannot be explained by adding word form information to instance-related information.

Both RSA and CA analyses were also conducted for the whole model architecture (six extrasylvian and six perisylvian model areas). The findings replicated previous results, indicating category learning (Extended Data Figure 4-1, Extended Data Table 4-1), generalization (Extended Data Figure 5-1, Extended Data Table 5-1), and representations of category critical as well as instance-specific features (Extended Data Figure 6-1, Extended Data Table 6-1).

## Discussion

When sensorimotor patterns simulating the processing of similar objects or actions from different categories were presented, the brain-constrained network applied in the current study showed successful conceptual category learning. Category learning outside symbol context was manifested in greater similarities of activity patterns elicited by different instances of the same category as compared with between-category pattern similarities. Importantly, compared with the training of instances per se, concurrent learning of category instances and symbols had a substantial effect on both categorial and instance-specific processes. Category term learning led to an additional increase in dissimilarities between activity patterns across conceptual categories, while making category members substantially more similar to each other. In contrast, proper name learning did not change between-category similarities and led to a relatively minor similarity increase between members of the same category. The model gave evidence of generalization to novel members of learned categories and showed that such generalization was maximal for novel members of categories for which CT had been learned. Meticulous analyses of neuronal activity patterns suggest that the enhancement of within-category similarities and between-category dissimilarities in the context of category symbols is due to an increase in the number of cells responding to all category members. Likewise, the relative persistence of instance-specific neurons with proper name learning underlies the maintained activation differences between category instances observed in this case. All observed effects regarding pattern dissimilarities and neuronal microstructure were greatly pronounced in the central “connector hub” areas of the brain-constrained model applied, as compared with primary areas. Table 3 summarizes major observations in the current data and the corresponding learning aspects these observations reflect.

**Table 3.** Critical and significant observations and the corresponding aspects of learning


Analysis	Learning aspect	Observation
RSA	Category learning	Successful category learning in all learning conditions $\mathrm{Dissi}m_{B-TT}>\mathrm{Dissi}m_{W-TT}$
		Interaction effect of symbol type and within/between categories $\mathrm{Dissi}m_{B-TT_{CT}}>\mathrm{Dissi}m_{B-TT_{PN}}$; $\mathrm{Dissi}m_{B-TT_{CT}}>\mathrm{Dissi}m_{B-TT_{PN}}$ $\mathrm{Dissi}m_{W-TT_{CT}}<\mathrm{Dissi}m_{W-TT_{PN}}$; $\mathrm{Dissi}m_{W-TT_{CT}}<\mathrm{Dissi}m_{W-TT_{NoL}}$
	Generalization	Symbol effect on dissimilarity differences within category $\mathrm{DissimDif}f_{CT}<\mathrm{DissimDif}f_{NoS}$ $\mathrm{DissimDif}f_{CT}<\mathrm{DissimDif}f_{PN}$
CA Analysis		Tendency to encode shared features in all learning conditions $n_{S}>n_{U}$
	Representations of category-critical features	Symbol effect on the number of shared neurons $n_{S_{CT}}>n_{S_{PN}}$; $n_{S_{CT}}>n_{S_{NoL}}$
		Gain in shared neurons in the central areas in all learning conditions $n_{S-\mathrm{central}}>n_{S-\mathrm{primary}}$
		Symbol effect on across-area gain of shared neurons $\mathrm{Gai}n_{S_{CT}}>\mathrm{Gai}n_{S_{PN}}$; $\mathrm{Gai}n_{S_{CT}}>\mathrm{Gai}n_{S_{NoL}}$
	Representations of instance-specific features	Symbol effect on the number of unique neurons $n_{U_{PN}}>n_{U_{CT}}$; $n_{U_{NoL}}>n_{U_{CT}}$
		Loss in unique neurons in the central areas in all learning conditions $n_{S-\mathrm{central}}>n_{S-\mathrm{primary}}$
		Symbol effect on across-area loss of unique neurons $\mathrm{Los}s_{S_{PN}}<\mathrm{Los}s_{S_{CT}}$

$\mathrm{Dissi}m_{W-TT}/\mathrm{Dissi}m_{W-TN}$, dissimilarity between a trained instance and another trained instance/novel instance of the same category; $\mathrm{Dissi}m_{B-TT}$, dissimilarity between two trained instances from different categories; $\mathrm{DissimDiff}=|\mathrm{Dissi}m_{W-TN}-\mathrm{Dissi}m_{W-TT}|$; $n_{S}$, number of shared neurons; $n_{U}$, number of unique neurons; CT, category term; PN, proper name; NoS, no symbol.

### Relationship to experimental and neurocomputational research

Our results can be used to address observations delivered by neurocognitive and neurobehavioral experiments. Neuropsychological evidence highlights the role of the prefrontal cortex in categorical representation (for review, see Kéri, 2003). Prefrontal areas (PF_i_ and PF_l_) are part of the four central areas of our model, where conceptual neurons constituting category representations emerged most numerously. This is explained by the high degree of convergence of neural activity in these areas, which are not only located in the center of the model architecture but also show the highest connectivity degrees. Due to ample activity converging on these connector hub areas, their frequently activated shared semantic neurons can most efficiently recruit other neurons, which therefore take on similar response properties (Doursat and Bienenstock, 2006). This mechanism may contribute to why these areas act as “semantic hubs” and house neurons reflecting category membership (e.g., PF and AT, see Miller et al., 2002; Seger and Miller, 2010; Garagnani and Pulvermüller, 2016; Tomasello et al., 2017). On the other hand, the higher density of instance-specific neurons in the primary visual/motor model area relative to the centre is evidence for exemplar learning in the sensorimotor cortices (Kéri, 2003; Bowman et al., 2020)—a type of category learning that is based on the representations of specific category instances (Nosofsky, 1988) and should be independent of signs and symbols. Here, solid evidence for category formation was obtained even in the control condition where only sensorimotor patterns were presented to the model without symbols. In line with neural data (Freedman et al., 2001; Seger and Miller, 2010), experimental evidence shows that perceptuomotor similarities among category members are sufficient to trigger category learning in preverbal infants (Sloutsky and Fisher, 2004; de Heering and Rossion, 2015) and animals (Güntürkün et al., 2018; Pusch et al., 2023).

When learning conceptual instances in the context of CT, infants show the most pronounced category building and an attention bias toward shared features of category members (Waxman and Markow, 1995; Dewar and Xu, 2007; Althaus and Mareschal, 2014). In contrast, encountering PN for individual instances focuses their attention relatively more on object-specific features (Barnhart et al., 2018; Pickron et al., 2018; La Tourette and Waxman, 2020). In the current network model, symbol association raises the number of neurons involved in the processing of a given sensorimotor pattern. This can be interpreted as biased attention to the object or action for which the pattern codes and thus explains why label learning generally increases attention to object features. Furthermore, as category term learning increases the number of category-critical shared semantic neurons in the network, at the cost of reducing the number of instance-specific ones, the preobserved greater attention to shared features has a direct model correlate, along with the label-related tendency to build stronger category representations. Infants’ attentional focus on instance-specific features of objects is in line with the relative preservation of instance-specific neurons in the model of proper name learning. Thus, the opposing effects of proper name and category term learning, which, respectively, drive attention toward instance-specific and category general features of objects, are captured by the current model.

A range of neurocomputational studies previously explored the putative brain basis of cognitive processes (Deco and Rolls, 2005; Rolls and Deco, 2015; Palm, 2016), including conceptual category learning and the influence of language on object perception (Rogers and McClelland, 2014; Henningsen-Schomers and Pulvermüller, 2022). For example, Westermann and Mareschal (2014) demonstrated, using a fully distributed parallel processing model, that learning a category label made the neural patterns of category members more similar to each other, whereas different categories moved away from each other in representational space. Our RSA in models mimicking cortical area structure and connectivity, along with within-area excitatory and inhibitory connectivity, achieved the same result. In addition, we determined the neuron-level mechanisms and contributions of different model areas to this result and, in particular, revealed the model–central connector hub areas as the loci where the differences between categorical and instance-specific mechanisms as well as those between the shared- versus specific-feature promoting roles of instance-specific and category labels are most pronounced. As to our knowledge, the contrast between activity patterns and neuronal correlates of PN and CT has not been addressed by previous computational work.

### Model explanation

The present simulations offer explanations of the observed phenomena based on neuroscience principles. Of special relevance here are the biological learning mechanisms applied, which include unsupervised Hebbian synaptic strengthening of connections between coactivated neurons and weakening of links between cells firing independently of each other. This principle explains why category labels primarily interlink with the shared neurons of instance representations belonging to the same category. The reason lies in the highest correlation values, as instance-specific neurons are silent when the category term is used together with other category instances. This implies some weakening of connections between the CT’ and the instance-specific neurons, based on the “anti-Hebbian” “neurons out-of-sync delink” rule. The opposite difference applies to PN, whose neural correlates strongly connect to instance-specific neurons but weaken their links with the category-critical shared neurons whenever a different category member co-occurs with its own and thus different name. Effects are most clearly present in the central areas of the network where the neural correlates of words and entities are equally manifest so that their correlation structure can easily be mapped.

### Limitations and future direction

The current simulations use idealized instance and category learning conditions. The activation patterns representing conceptual instances and word forms were chosen to be nonoverlapping, except for the neurons coding for shared features. These are idealizations considering both the features of word forms and those of objects and actions could be shared across categories (compare phonological, e.g., “cat”-“hat” or perceptual color/shape similarities). Such similarities are irrelevant to category membership and hence were omitted to keep the simulation well-controlled. Secondly, only a small number of conceptual features were realized, and a small set of shared features determined concept membership. This situation may hold for some concrete terms but not for others and certainly not for abstract concepts (Henningsen-Schomers et al., 2022). Furthermore, PN and CT were acquired by different networks to allow straightforward separation and evaluation of the mechanistic side of different label types—although label types are normally copresent in the same mind and brain. In the future, it is desirable to complement this work with simulations of more realistic conceptual categories and to build one model in which interaction/interference effects between different learning conditions are possible.

## Conclusion

The current study strived to meet the need for a mechanistic model of symbols and their meaning within a neurobiological computational framework by addressing specific features of PN (Mickey Mouse) and category symbols (house mouse). Developmentalists and linguists have long been proposing that CT and PN distinctively impact infants’ locus of attention toward category-shared and instance-specific object and action features, respectively. By simulating concept and instance learning in a deep neural network with neurobiologically realistic architecture and brain-like connectivity, we demonstrate that learning these two different symbol types had opposing effects on the emergent neuronal CAs representing and processing instances of a category and the shared conceptual features of that category, which can explain preobserved differences in perceptual, attentive, and memory processes related to the specific and shared features of category instances. These explanations were based on unsupervised Hebbian associative learning mechanism binding neurons involved in correlated processing of instance-specific category general information. The current work could thus not only replicate but also offer underlying neuronal mechanisms and causal neurobiological explanations for well-established observations in cognitive science.
