# Supplementary figures and images for: Causal Influence of Linguistic Learning on Perceptual and Conceptual Processing: A Brain-Constrained Deep Neural Network Study of Proper Names and Category Terms
Source: J Neurosci. 2024 Feb 28;44(9):e1048232023. doi: 10.1523/JNEUROSCI.1048-23.2023 (PMC10904026; doi:10.1523/JNEUROSCI.1048-23.2023)

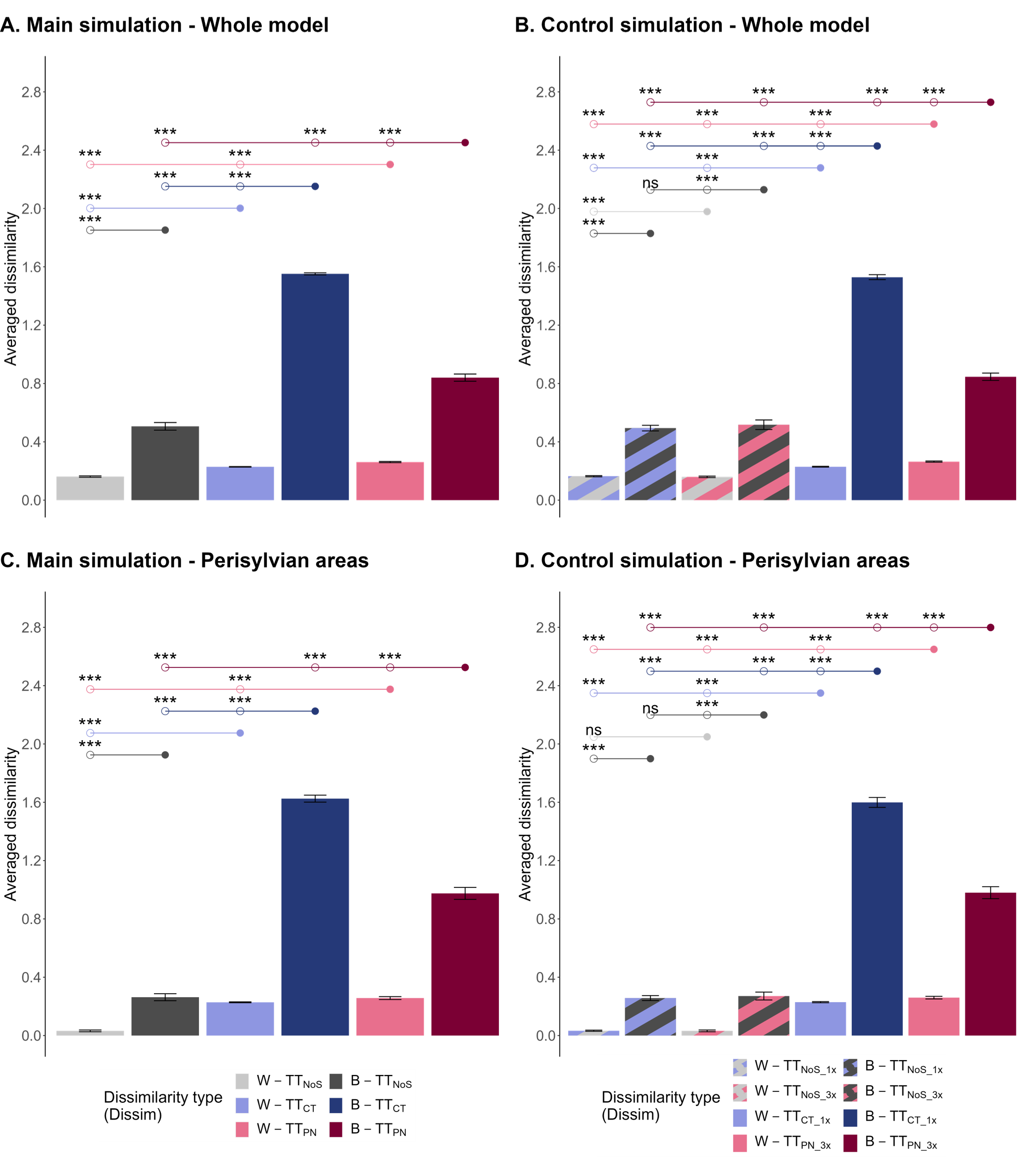

Supplement: Figure 4-1 — Bar charts depicting dissimilarities between network activity elicited by trained grounding patterns after learning for each of the three training conditions. Within-category (W-TT) and between-category (B-TT) dissimilarities across the 30 trained items were averaged for A-B) all 12 model areas and C-D) extrasylvian model areas. For further explanation, see Figure 4. Download Figure 4-1, TIF file. [file jneuro-44-e1048232023-s001.tif]

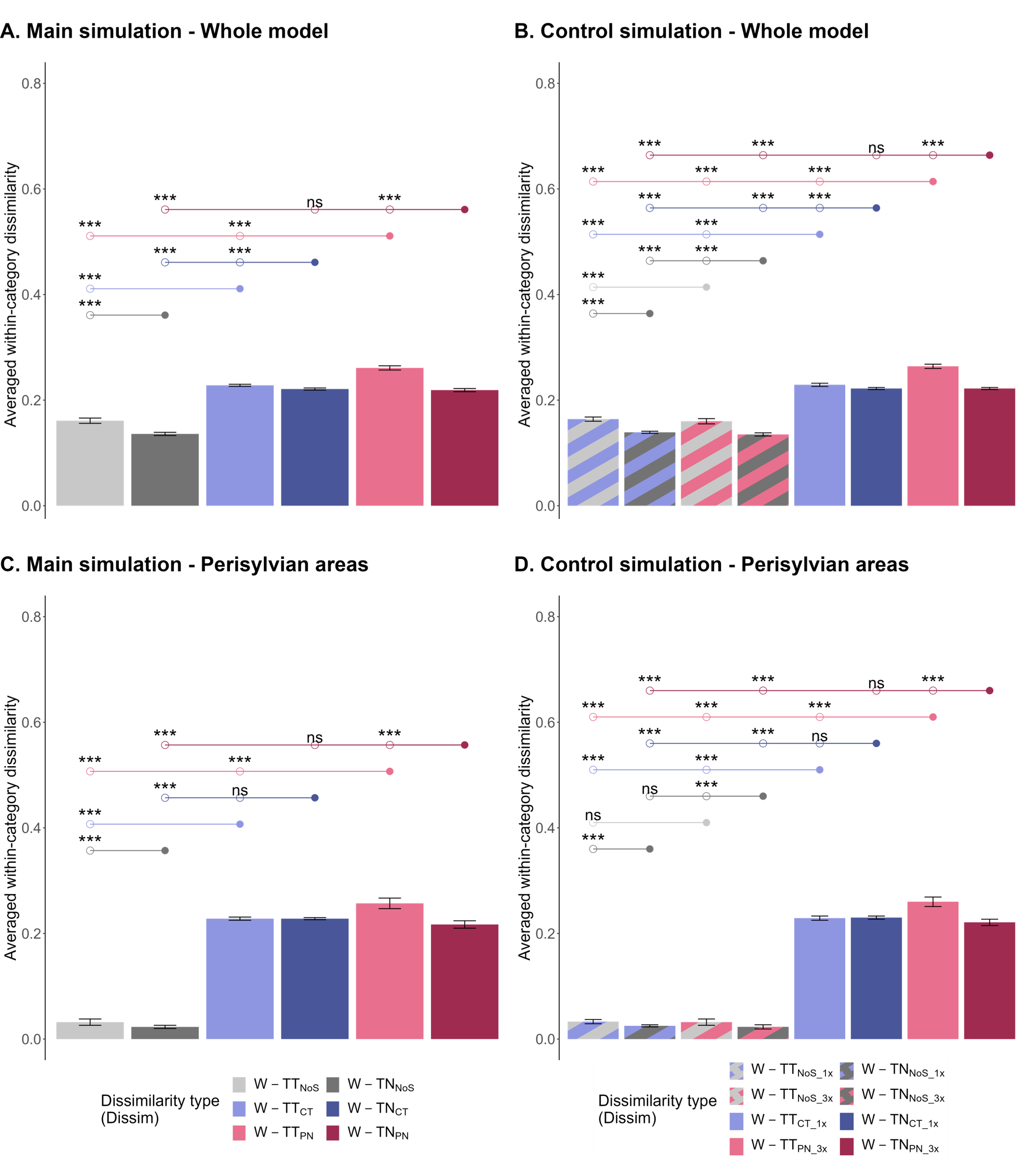

Supplement: Figure 5-1 — Bar charts depicting dissimilarities between network activity areas elicited by trained novel grounding patterns after learning for each of the three training conditions. Within-category dissimilarities between any two trained instances (W-TT) and between trained and novel instances (W-TN) were averaged for A&B) all 12 model areas and C&D) perisylvian model areas. For further explanation, see Figure 4. Download Figure 5-1, TIF file. [file jneuro-44-e1048232023-s002.tif]

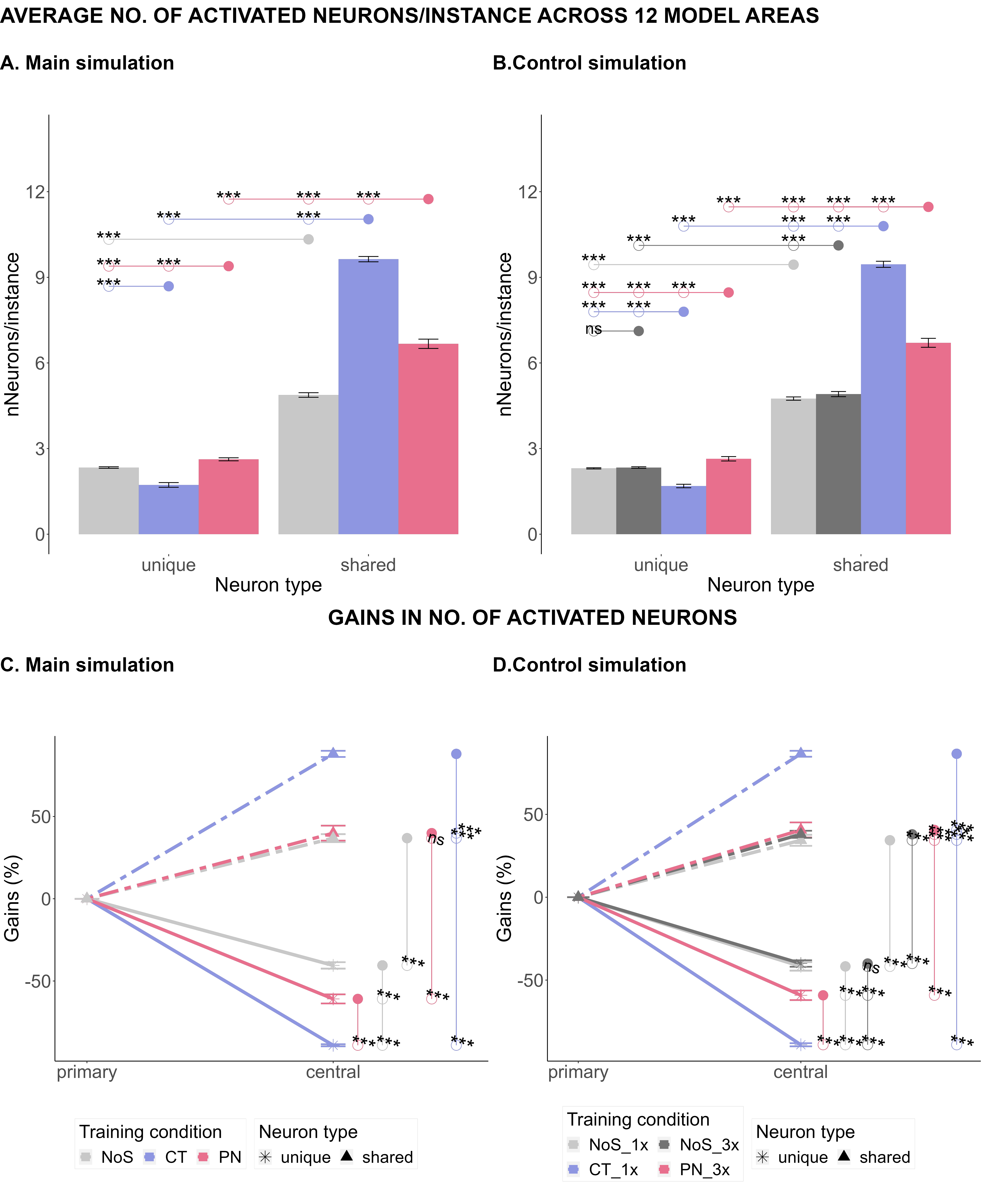

Supplement: Figure 6-1 — A &B) The number of activated neurons in response to the 30 trained grounding patterns was averaged for each of the 12 model areas. D&E) Changes in neuronal activation seen between primary areas, where stimulation was given, and the ‘higher’ more central connector hub areas central to the architecturssssse. For further explanations see Figure 4. Download Figure 6-1, TIF file. [file jneuro-44-e1048232023-s003.tif]
