# Supplementary material for: Causal Influence of Linguistic Learning on Perceptual and Conceptual Processing: A Brain-Constrained Deep Neural Network Study of Proper Names and Category Terms
Source: J Neurosci. 2024 Feb 28;44(9):e1048232023. doi: 10.1523/JNEUROSCI.1048-23.2023 (PMC10904026; doi:10.1523/JNEUROSCI.1048-23.2023)
Supplement: Table 4-1 — ANOVA table reporting significant effects of training condition (No symbol/Category term/Proper name) and dissimilarity type (DissimW−TT/DissimB−TT) on averaged dissimilarity across 12 model areas. Download Table 4-1, DOCX file. [file jneuro-44-e1048232023-s004.docx]

|  | ***Df*** | ***F*** | $\boldsymbol{\eta}^{\boldsymbol{2}}$ |
| --- | --- | --- | --- |
| Training condition (TC) | 2 | 4162.999*** | 0.986 |
| Dissimilarity type (DT) | 1 | 17585.345*** | 0.993 |
| TC$\times$DT | 2 | 3754.906*** | 0.982 |
