# Supplementary material for: Causal Influence of Linguistic Learning on Perceptual and Conceptual Processing: A Brain-Constrained Deep Neural Network Study of Proper Names and Category Terms
Source: J Neurosci. 2024 Feb 28;44(9):e1048232023. doi: 10.1523/JNEUROSCI.1048-23.2023 (PMC10904026; doi:10.1523/JNEUROSCI.1048-23.2023)
Supplement: Table 6-1 — ANOVA table reporting significant effects (across 12 area model) of training condition (No symbol/Category term/Proper name) and neuron type (shared/unique) on the number of activated neurons (left) and on the gain/loss of unique/shared neurons from primary areas to the connector hub (right). Download Table 6-1, DOCX file. [file jneuro-44-e1048232023-s006.docx]

|  | ***nNeuron/instance*** | | | **Gain (%)** | | |
| --- | --- | --- | --- | --- | --- | --- |
|  | ***Df*** | ***F*** | $\boldsymbol{\eta}^{\boldsymbol{2}}$ | ***Df*** | ***F*** | $\boldsymbol{\eta}^{\boldsymbol{2}}$ |
| Training condition (TC) | 2 | 824.067*** | 0.969 | 2 | 55.178*** | 0.551 |
| Neuron type (NT) | 1 | 7842.134*** | 0.986 | 1 | 6471.541*** | 0.995 |
| TC$\times$NT | 2 | 997.741*** | 0.957 | 2 | 1484.439*** | 0.966 |
| *Df*: degree of freedom. ***p<0.001 | | | | | | |
